# Nonspecific chest pain and hospital revisits within 7 days of care: variation across emergency department, observation and inpatient visits

**DOI:** 10.1186/s12913-020-05200-x

**Published:** 2020-06-08

**Authors:** Grant R. Martsolf, Teryl K. Nuckols, Kathryn R. Fingar, Marguerite L. Barrett, Carol Stocks, Pamela L. Owens

**Affiliations:** 1 University of Pittsburgh School of Nursing, 3500 Victoria St, 315B, Pittsburgh, PA 15213 USA; 2 RAND Corporation, 4570 Fifth Ave #600, Pittsburgh, PA 15213 USA; 3 RAND Corporation, 1776 Main Street, Santa Monica, CA 90401 USA; 4 Cedars-Sinai Medical Center, 8700 Beverly Boulevard, Becker 113, Los Angeles, CA 90048 USA; 5 IBM Watson Health, 5425 Hollister Ave, Suite 140, Santa Barbara, CA 93111 USA; 6 ML Barrett, Inc., 13943 Boquita Drive, Del Mar, CA 92014 USA; 7 Affiliation during this investigation: Agency for Healthcare Research and Quality, Rockville, Maryland USA; 8 Present address: West Virginia University, School of Public Health, 64 Medical Center Drive, PO Box 9190, Morgantown, WV 26506-9190 USA; 9 Agency for Healthcare Research and Quality, 5600 Fishers Lane, Rockville, MD 20857 USA

**Keywords:** Chest pain, Hospital, Readmission, Observation services, Acute care, Emergency department, Insurance

## Background

Patients increasingly receive care in outpatient observation services rather than being admitted to the hospital, and chest pain is the leading diagnosis among observed patients [1–4]. Chest pain can indicate a serious medical condition (e.g., acute coronary syndromes, including acute myocardial infarction [AMI] and unstable angina) or a relatively minor one (e.g., gastroesophageal reflux). Often the difference between a serious and minor condition is not readily apparent [5, 6]. A relatively small percentage of patients who present to the hospital with chest pain have acute coronary syndromes or other emergent conditions requiring inpatient care [7]. However, determining the diagnosis and appropriate course of treatment for chest pain can involve clinical evaluation, testing, and monitoring. Clinicians historically had two options: hold patients with chest pain in the emergency department (ED), or admit them to the hospital for a short stay. Observation status offers a third option, allowing more time to assess the patient’s condition.

The concept of hospital observation emerged in the 1970s as an alternative to ED and inpatient care [8, 9]. For chest pain, randomized controlled trials in the 1990s and 2000s demonstrated that dedicated cardiac observation units and specific care protocols improved outcomes, reduced hospital admissions, and lowered costs [10–13]. The practice of hospital observation status has evolved over time, and expanded in diverse ways across different states and payers. Many public and private payers now reimburse observation services as a hospital-based, outpatient service irrespective of diagnosis, use of a specific protocol, or physical location in the hospital [14, 15]. The Centers for Medicare & Medicaid Services defines observation services as “a well-defined set of specific, clinically appropriate services, which include ongoing short term treatment, assessment, and reassessment before a decision can be made regarding whether patients will require further treatment as hospital inpatients or if they are able to be discharged from the hospital.” [16]

Despite rapid growth of observation services for chest pain [17, 18], it is unclear whether observation services in current practice is associated with different outcomes compared with ED or inpatient care [19]. Unlike the historical randomized controlled trials previously mentioned, many hospitals do not have designated observation units or use chest pain protocols [18]. A potentially useful outcome measure following an ED, observation, or inpatient hospital visit is the percentage of patients who return to the hospital [20, 21]. Compared with longer follow-up periods, a 7-day follow-up period emphasizes revisits that are more likely to involve acute issues related to the index visit [22, 23]. Moreover, the first week after a hospital visit for chest pain represents a high-risk period during which atherosclerotic heart disease and AMI are leading causes of death [23, 24]; thus, return visits for acute coronary syndromes are particularly concerning.

Our primary objective was to compare the rate at which patients with nonspecific chest pain return to the hospital within 7 days after index observation visits versus after index ED and inpatient visits. Although acuity likely differs across these three types of visits, no benchmarks have been established for assessing outcomes of observation care, making ED and inpatient care useful points of comparison [25]. Secondary objectives included examining [1] the type of revisit (ED, observation, or inpatient) to determine how often patients receive a higher level of care upon return and [2] first-listed diagnoses at the revisit to assess whether patients may be returning for acute coronary syndromes, nonspecific and other causes of chest pain, or other reasons.

## Methods

### Study data

We used 2013 and 2014 data from the Healthcare Cost and Utilization Project (HCUP) [26] State Inpatient Databases (SID), State Emergency Department Databases (SEDD), and State Ambulatory Surgery and Services Databases (SASD) [27]. We selected 10 states (Georgia, Iowa, Maryland, Nebraska, Nevada, South Carolina, South Dakota, Tennessee, Vermont, and Wisconsin) because they had complete data on observation services and encrypted patient linkage numbers that linked index visits and revisits within the same state. These states represented 13% of the U.S. population in 2014 (4% West, 23% South, 17% Midwest, and 1% Northeast).

### Study population

We included patients aged 18 years or older with an index visit for a first-listed (or principal) diagnosis of nonspecific chest pain. The unit of analysis was an index visit—ED visit, short-stay observation visit, or short-stay inpatient visit (defined below). Patients with multiple encounters contributed multiple index visits if the index visit met the following exclusion and inclusion criteria. We excluded index visits if the patient transferred into or out of the hospital, died in the hospital, or left against medical advice. We required that index visits have a clean period, defined as no hospitalization, observation visit, or ED visit for any reason in the past 30 days [28]. To identify events that occurred before and after the index visit, we only included index visits that occurred between February 2013 and November 2014.

### Independent variable

The primary independent variable was the type of index visit for nonspecific chest pain:

1. *Index ED visit*. The patient was discharged from ED care without being placed under observation or admitted.
2. *Index observation visit*. The patient was discharged from observation care within 2 nights after presentation to the hospital without being admitted.
3. *Index inpatient visit.* The patient was discharged from short-stay inpatient care within 2 nights after presentation to the hospital.

Determination of type of index visit was based on status at time of discharge (e.g., patients seen in the ED but then moved to observation were considered having an observation visit. We included only short-stay observation and inpatient visits (discharge within 2 nights [15, 29] after presentation to the hospital) to increase the comparability of the three types of visits.

### Dependent variables

The primary dependent variable was the presence or absence of any revisit to the hospital—meaning ED, observation, or inpatient visits—within 7 days of the discharge date from the index visit. Secondary dependent variables included type of care (ED, observation, or inpatient) and diagnosis at the revisit. For the latter, we created a categorical variable based on the following hierarchy: (1) any diagnosis of acute coronary syndromes (AMI or unstable angina), (2) any diagnosis of nonspecific chest pain and other conditions potentially associated with chest pain (e.g., other cardiac conditions, lower respiratory disease, esophageal disorders), and (3) unrelated conditions (defined in Additional file 1 Table S1) [5].

### Covariates

Covariates included patient sex, age, expected primary payer (Medicare, Medicaid, private, self-pay / no charge), urban/rural location, median household income quartile (derived using patient ZIP Code), as well as coexisting conditions at the index visit: prior AMI, any other ischemic heart disease, other comorbid conditions identified using Elixhauser Comorbidity software [30], designed to predict 30-day readmissions. We included 14 out of 29 comorbid conditions, which represented at least 1% of all index records in all three settings. We also included a variable representing the number of diagnoses reported on the record, because coexisting conditions may be documented more frequently for inpatient and observation than for ED visits.

### Analysis

We estimated separate logistic regression models for the primary and secondary dependent variables including the covariates described above and hospital-level fixed effects, because care may be influenced by unknown factors that are constant across visits to a particular hospital.

We used results from the models to derive risk-adjusted 7-day revisit rates for different types of index visits, applying a widely used approach for risk adjustment (described in the Additional file) [31]. We repeated these analyses stratifying by expected primary payer, because insurance is likely to influence access to ambulatory care after a hospital visit. We also repeated these analyses using the secondary outcome of diagnosis at the revisit. Because large sample sizes increase the probability of a type I error, we considered results statistically significant if the *p*-value was < 0.01 and the revisit rates differed by 10% or more.

## Results

### Sample characteristics

We included 1,005,866 index visits to 746 hospitals for nonspecific chest pain. Most index visits occurred in the ED (638,302) with about half as many index observation visits (325,770) and fewer index inpatient visits (41,794). Compared with patients with index observation visits, patients with index inpatient visits were more likely to be older, have Medicare insurance, and have more documented coexisting conditions, whereas patients with index ED visits were more likely to be younger, have private insurance, and have fewer coexisting conditions (Table 1).

**Table 1:** Nonspecific Chest Pain Index Visit Characteristics, by Type of Index Visit, 2013 and 2014

Characteristic	Type of Index Visit		
	ED	Observation	Inpatient
Total, N	638,302	325,770	41,794
Originated in the ED	638,302	313,000	37,363
Direct admission	N/A	12,770	4431
Female, %	57.8	57.0	52.8
Age, years, mean	46.6	57.9	60.0
Expected primary payer, %			
Medicare	23.7	38.7	47.3
Medicaid	17.1	10.7	10.3
Private	36.3	35.3	27.5
Self-pay / No charge	18.8	11.5	10.5
Other	3.9	3.5	4.2
Patient’s income quartile, %			
Quartile 1 (poorest)	35.5	34.6	35.9
Quartiles 2 and 3	49.5	47.7	48.8
Quartile 4 (wealthiest)	13.6	16.2	13.7
Location of residence, %			
Large metropolitan	37.7	42.7	42.4
Small metropolitan	39.6	34.6	36.2
Micropolitan	12.7	12.6	12.7
Rural	9.8	9.9	8.5
Diagnoses reported on the index visit, mean N	3.9	8.3	10.4
Most frequent coexisting condition, %^a^			
Elixhauser Comorbidities^b^			
Alcohol abuse	1.4	2.7	4.7
Chronic pulmonary disease	8.8	16.8	21.6
Congestive heart failure	2.5	5.9	12.0
Deficiency anemias	1.4	5.6	11.0
Depression	3.2	10.7	13.0
Diabetes	10.7	25.5	33.1
Drug abuse	1.9	2.9	4.4
Fluid/electrolyte disorders	2.3	9.6	15.5
Hypertension	29.9	65.6	75.6
Hypothyroidism	2.4	10.3	12.2
Obesity	2.2	14.6	19.9
Other neurological disorders	1.7	4.4	6.3
Psychoses	1.6	3.4	4.7
Renal failure	1.5	5.3	12.4
Related to heart disease			
Other ischemic heart disease^c^	6.3	24.4	41.2
Prior AMI	3.4	8.5	13.7
Revisits, N	62,629	20,472	3591

Abbreviations: *AMI* Acute myocardial infarction, *ED* Emergency department, *N* Number, *N/A* Not applicable

Note: Percentages may not sum to 100% because some index visits are missing the information

^a^Coexisting conditions include those from the Elixhauser comorbidity software and those related to heart disease. Only conditions with at least 1% of records in all three settings are shown. The diabetes measure includes diabetes with and without complications

^b^The following indicators were defined using the Elixhauser Comorbidity Software, the specifications of which can be found at https://www.hcup-us.ahrq.gov/toolssoftware/comorbidity/comorbidity.jsp

^c^Defined using the ICD-9 CM code 414, Other forms of chronic ischemic heart disease

Source: Agency for Healthcare Research and Quality, Healthcare Cost and Utilization Project, State Inpatient Databases, State Emergency Department Databases, and State Ambulatory Surgery and Services Databases, 10 states (Georgia, Iowa, Maryland, Nebraska, Nevada, South Carolina, South Dakota, Tennessee, Vermont, Wisconsin), 2013 and 2014

**Fig. 1:**
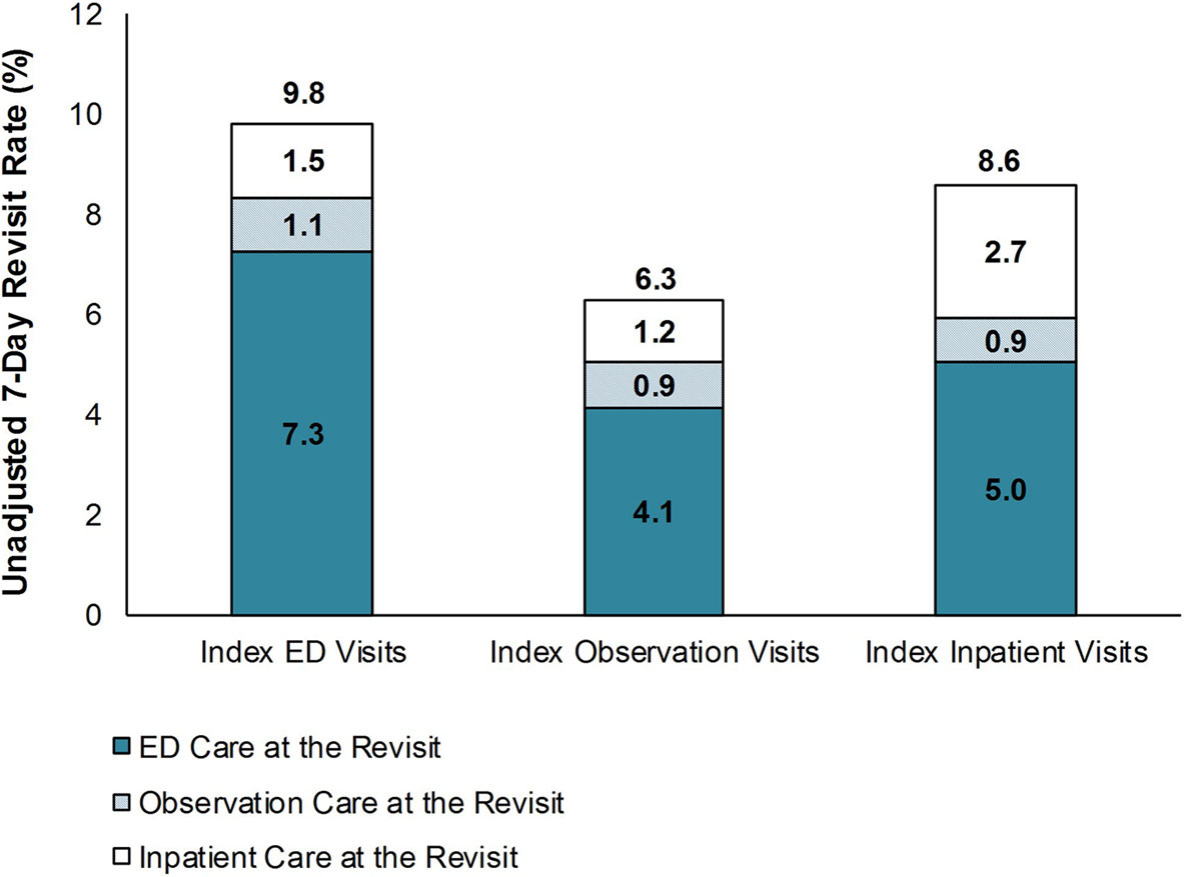
Unadjusted 7-Day Revisit Rate, by Type of Index Visit, 2013 and 2014. Abbreviation: ED, emergency department. Source: Agency for Healthcare Research and Quality, Healthcare Cost and Utilization Project, State Inpatient Databases, State Emergency Department Databases, and State Ambulatory Surgery and Services Databases, 10 states (Georgia, Iowa, Maryland, Nebraska, Nevada, South Carolina, South Dakota, Tennessee, Vermont, Wisconsin), 2013 and 2014

There were 86,692 revisits (62,629 following index ED visits, 20,472 following index observation visits, and 3591 following index inpatient visits). Total unadjusted 7-day revisit rates after index visits for ED care, observation care, and inpatient care were 9.8, 6.3, and 8.6%, respectively (Fig. 1). Regardless of the type of index visit, over half of 7-day revisits had been discharged from the ED without observation or inpatient care. The rates at which patients returned for inpatient care were 1.5% among patients with an index visit for ED care, 1.2% among patients with index observation visits, and 2.7% among patients with index inpatient visits.

### Seven-day risk-adjusted revisit rate

**Fig. 2:**
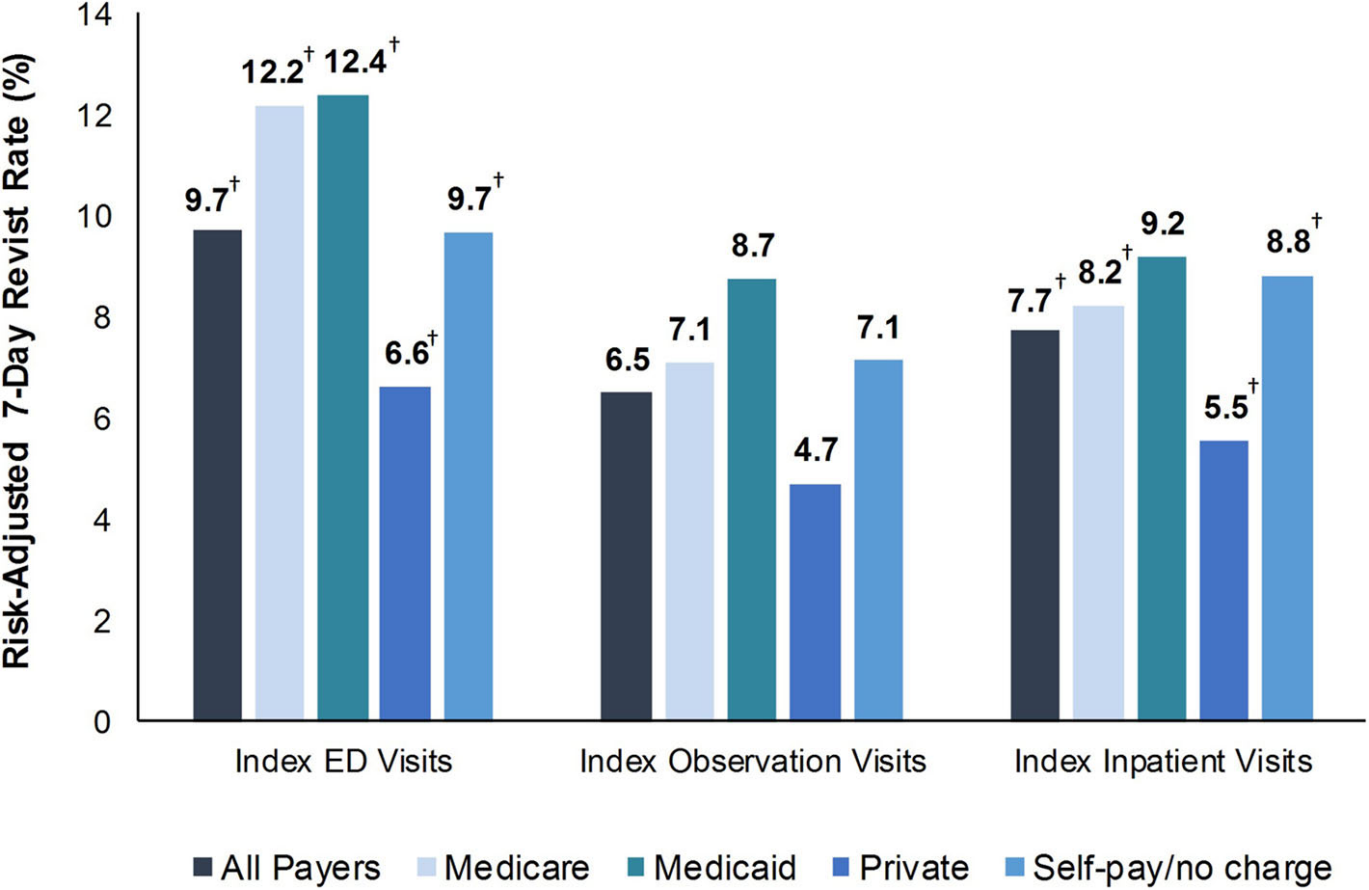
Risk-Adjusted 7-Day Revisit Rate, by Payer Population and Type of Index Visit, 2013 and 2014. Abbreviation: ED, emergency department. ^†^ Statistically significant at *p* < 0.05 and ≥ 10% difference between revisit rates for index observation visits and index ED or inpatient visits. Source: Agency for Healthcare Research and Quality, Healthcare Cost and Utilization Project, State Inpatient Databases, State Emergency Department Databases, and State Ambulatory Surgery and Services Databases, 10 states (Georgia, Iowa, Maryland, Nebraska, Nevada, South Carolina, South Dakota, Tennessee, Vermont, Wisconsin), 2013 and 2014

Adjusted revisit rates following an index visit for nonspecific chest pain involving ED care, observation care, and inpatient care were 9.7% (95% CI: 9.7–9.8), 6.5% (95% CI: 6.4–6.6), and 7.7% (95% CI: 7.5–8.0), respectively (Fig. 2). The risk-adjusted 7-day revisit rate was 3.2 (95% CI: 3.1–3.3) and 1.2 (95% CI, 1.0–1.5) percentage points lower, respectively, following an index observation visit than following an index ED (*p* < 0.001) or inpatient (*p* < 0.001) visit.

### Revisit rates by payer population

Results were similar across payers (Fig. 2). Relative to the risk-adjusted 7-day revisit rate following an index ED visit, the rate following an index observation visit was 5.1 (95% CI: 4.9–5.3) percentage points lower among patients with Medicare (*p* < 0.001), 3.7 (95% CI: 3.3–3.9) percentage points lower among patients with Medicaid (*p* < 0.001), 1.9 (95% CI: 1.7–2.1) percentage points lower among patients with private insurance (*p* < 0.001), and 2.6 (95% CI: 2.2–2.8) percentage points lower among patients with no insurance (*p* < 0.001). Relative to the risk-adjusted 7-day revisit rate following an index inpatient visit, the rate following an index observation visit was 1.1 (95% CI: 0.7–1.5) percentage points lower among patients with Medicare (*p* < 0.001), 0.8 (95% CI: 0.3–1.4) percentage points lower among patients with private insurance (*p* < 0.01), and 1.7 (95% CI: 0.9–2.5) percentage points lower among patients with no insurance (*p* < 0.001). Among patients with Medicaid, revisit rates were similar after index visits for observation and inpatient care.

### Diagnosis at revisit

**Fig. 3:**
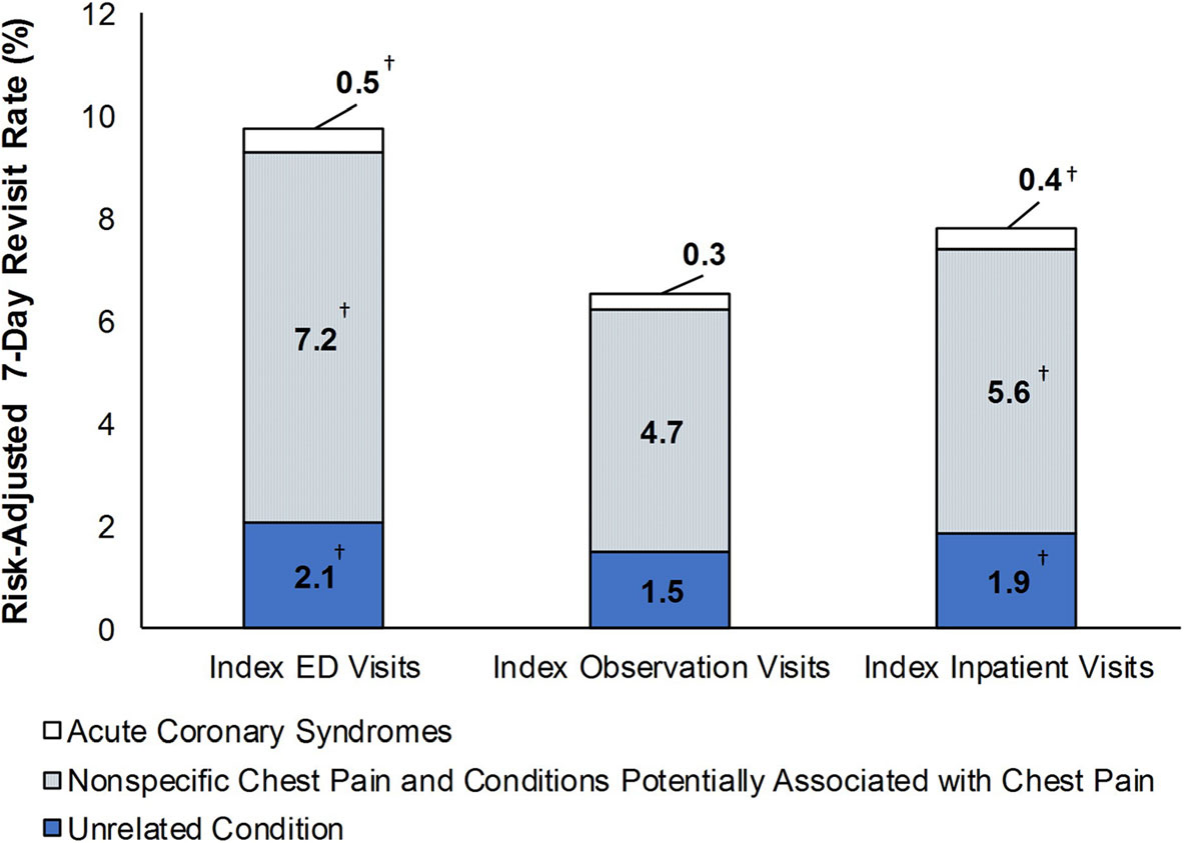
Diagnosis at Revisit: Risk-Adjusted 7-Day Revisit Rates by Type of Index Visit, 2013 and 2014. Abbreviation: ED, emergency department. ^†^ Statistically significant at *p* < 0.05 and ≥ 10% difference between revisit rates for index observation visits and index ED or inpatient visits. Source: Agency for Healthcare Research and Quality, Healthcare Cost and Utilization Project, State Inpatient Databases, State Emergency Department Databases, and State Ambulatory Surgery and Services Databases, 10 states (Georgia, Iowa, Maryland, Nebraska, Nevada, South Carolina, South Dakota, Tennessee, Vermont, Wisconsin), 2013 and 2014

Revisits for acute coronary syndromes were rare (0.3 to 0.5% of index visits), while revisits for nonspecific chest pain and conditions potentially associated with chest pain were common (4.7 to 7.2% of index visits), irrespective of the type of index visit (Fig. 3). The risk-adjusted rate at which patients returned within 7 days with acute coronary syndromes was 0.2 (95% CI: 0.1–0.2) and 0.1 (95% CI: 0.0–0.2) percentage points lower, respectively, after an index observation visit than after an index ED visit (*p* < 0.001) and after an index inpatient visit (*p* < 0.001). Additionally, the risk-adjusted rate at which patients returned within 7 days with nonspecific chest pain or conditions potentially associated with chest pain were 2.5 (95% CI: 2.4–2.6) and 0.9 (95% CI: 0.6–1.1) percentage points lower, respectively, after an index observation visit than after an index ED (*p* < 0.001) or inpatient (*p* < 0.001) visit.

Patients most frequently returned to the hospital with nonspecific chest pain or conditions potentially associated with chest pain. The risk-adjusted 7-day revisit rate following an index ED visit was 9.7% (95% CI: 9.7–9.8), including a 7.2% (95% CI: 7.2–7.3) revisit rate for nonspecific chest pain or conditions potentially associated with chest pain (i.e., 74.3% of total revisits). The results from Fig. 3 were largely consistent across payers (not shown).

## Discussion

The present analysis of hospital-based visits for nonspecific chest pain has two notable findings. First, nearly 1 in 10 patients discharged from the ED and 1 in 13 patients discharged following an inpatient hospitalization returned to the hospital within 7 days of the index visit. Second, compared with ED and inpatient visits, observation visits for nonspecific chest pain were associated with lower revisit rates (1 in 15).

Risk stratification for acute coronary syndromes is a critical task in the evaluation of patients with chest pain. Reassuringly, very few returning patients in our analysis (< 0.5%) had acute coronary syndromes, suggesting that prevailing hospital practices effectively identify patients at low short-term risk. These results are consistent with a recent clinical trial [32]. In that study, patients presenting to the ED with acute chest pain, non-ischemic electrocardiograms, and negative biomarkers were at low risk of major adverse cardiac events in the subsequent month whether or not they underwent non-invasive stress testing [32].

Although patients rarely returned with acute coronary syndromes, we found that many came back with nonspecific chest pain or conditions associated with chest pain. The high rate of 7-day revisits for these recurrent, uncontrolled symptoms raise concerns—for reasons similar to the problems associated with high 30-day readmission rates for a variety of conditions following inpatient hospitalization. Hospital revisits represent potentially avoidable healthcare utilization, they may contribute to ED and hospital overcrowding, and, most importantly, they demonstrate that patients have unmet needs for care. The frequency of revisits raises questions about healthcare delivery factors including the quality of care at the index visit, the transition from the index visit to follow-up care, and access to outpatient care after the index visit. In addition to healthcare delivery factors, diverse patient factors may be associated with 7-day revisit rates including age, comorbidity, income, health insurance, social supports, health literacy, self-efficacy in managing symptoms, language barriers, and other social determinants of health.

Another factor potentially contributing to revisit rates is that clinical standards of care for nonspecific chest pain remain poorly defined. Clinical practice guidelines emphasize ruling out acute coronary syndromes and other acute life-threatening conditions, but provide less information on how to evaluate and manage nonspecific and noncoronary causes of chest pain [5, 33]. After acute coronary syndromes have been excluded, a comprehensive work-up often reveals that patients with nonspecific chest pain have gastroesophageal or chest wall conditions [34]. One concern of note is that the one-year mortality among patients with nonspecific chest pain is similar to that of patients with acute coronary syndromes, and 40% of patients with nonspecific chest pain have underlying coronary heart disease [35]. Thus, future research is needed to refine clinical standards of care for this population beyond the current focus on ruling out acute coronary syndromes. Furthermore, in addition to clinical decision-making, understanding the role of the healthcare delivery system and patient factors on revisit rates is important. For example, the decision to place patients with chest pain under observation can also be influenced by the availability of inpatient beds or whether family support is available if the patient were to be discharged.

Although originally designed to avoid unnecessary inpatient hospitalizations, observation care may allow clinicians a greater opportunity to diagnose noncardiac causes of chest pain and to educate patients in the self-management of symptoms. We found that, relative to ED visits, 7-day revisit rates were 3.2 percentage points lower (˗33.0%) across all populations and 5.1 percentage points lower (− 41.8%) among Medicare patients following index observation visits. Given our study design, these differences could be due to selection bias in the populations placed under observation vs. discharged from the ED. However, our findings were similar with and without adjustment, across payer populations, and by diagnosis at the return visit. Moreover, observed patients were older than those discharged from the ED, suggesting that they would be expected to have higher, not lower, revisit rates.

The results of our study are similar to two previous studies that examined outcomes after hospital visits for chest pain among Medicare beneficiaries [36] and patients within the Veteran’s Affairs system [29]. Both studies found that patients cared for in observation were 7–12% less likely to return to the hospital than those who received inpatient care. The study of Medicare beneficiaries found a 29% reduction in the risk of readmission for observed patients compared with those cared for in the ED. [36] These studies focused on 30-day revisits as opposed to 7-day revisits, so we cannot directly compare results. Our study extends the literature by including all adult patients and payers (including self-pay/no charge). We found similar patterns across payer populations, though the differences in revisit rates between observation and ED care were largest for Medicare patients.

Our study has several limitations. First, coding for coexisting conditions likely differs across ED, observation, and inpatient visits. For example, obesity was coded more often during inpatient and observation visits than during ED visits. We re-ran our models with and without conditions for which documentation varied across types of visits, as well as with and without the number of secondary conditions listed on the record, and results were similar. Second, risk-adjusted revisit rates may be higher for inpatient care than observation care because of differences in severity that are not observable in our dataset. For instance, claims data lack clinical information, such as positive cardiac biomarkers, which would reflect the risk of acute coronary syndromes. We were unable to identify revisits for patients who sought care in states not included in this study and we were unable to link records from patients who visited hospitals in multiple states. Finally, the fact that placing some patients under observation is associated with lower revisit rates does not imply that more patients with chest pain should be placed under observation in the future. Rather, the high revisit rates for nonspecific chest pain warrant thorough investigation, including rigorous testing of any future interventions designed to reduce revisits.

## Conclusion

In conclusion, up to 1 in 10 patients discharged with nonspecific chest pain returned to the hospital within 1 week. Compared with ED and inpatient care, observation visits were associated with lower revisit rates. The diagnoses at revisits were largely nonspecific chest pain and conditions associated with chest pain; acute coronary syndromes were very rare. The high rates of revisits demonstrate that patients may have unmet needs for care going beyond the need to rule out acute coronary syndromes. Future research is needed to refine clinical standards of care for nonspecific chest pain as well as investigate the healthcare delivery and patient factors that influence 7-day revisit rates.

## Supplementary information

**Additional file 1.Appendix Table S1.** Risk Adjustment Method and Conditions at the Revisit by Type of Index Visit.

## Data Availability

The intramural datasets generated and/or analyzed during the current study are not publicly available. However, public-use State Inpatient Databases (SID), State Emergency Department Databases (SEDD), and State Ambulatory Surgery and Services Databases (SASD) are available through the Healthcare Cost and Utilization Project Central Distributor: https://www.hcup-us.ahrq.gov/databases.jsp.

## Abbreviations

AHRQ: Agency for Healthcare Research and Quality
AMI: Acute myocardial infarction
CCS: Clinical Classifications Software
CI: Confidence interval
ED: emergency department
HCUP: Healthcare Cost and Utilization Project
N: Number
N/A: Not applicable
SASD: State Ambulatory Surgery and Services Databases
SEDD: State Emergency Department Databases
SID: State Inpatient Databases
