# Supplementary material for: Nonspecific chest pain and hospital revisits within 7 days of care: variation across emergency department, observation and inpatient visits
Source: BMC Health Serv Res. 2020 Jun 8;20:516. doi: 10.1186/s12913-020-05200-x (PMC7278151; doi:10.1186/s12913-020-05200-x)
Supplement: Supplementary file 1 — Additional file 1.Appendix Table S1. Risk Adjustment Method and Conditions at the Revisit by Type of Index Visit. [file 12913_2020_5200_MOESM1_ESM.docx]

**APPENDIX**

**Risk Adjustment Methods**

Steps to calculate a risk-adjusted 7-day revisit rate for each type of index visit (e.g., for index ED visits) involved multiplying the crude revisit rate across all index stays by the ratio of the observed to expected number of revisits. First, we used the regression model to predict each individual index visit’s risk-adjusted probability of having any revisit, using the covariates described above. Second, we summed the predicted probabilities across all index visits of that type (e.g., across all index ED visits) to determine the expected number of revisits. Finally, we divided the observed by the expected number of revisits and multiplied this quotient by the crude revisit rate to derive the risk-adjusted revisit rate.

**Appendix Table 1. Conditions at the Revisit by Type of Index Visit**

| **Category** | **Type of Index Visit** | | |
| --- | --- | --- | --- |
|  | **ED** | **Observation** | **Inpatient** |
| **Acute Coronary Syndromes (International Classification of Diseases, Ninth Revision, Clinical Modification code may be listed as any diagnosis), % of revisits** | | | |
| 410.x0, 410.x1: Acute myocardial infarction | 1.7 | 1.9 | 2.2 |
| 411.1: Unstable/accelerated/crescendo angina | 1.6 | 2.7 | 2.5 |
| 413.0: Nocturnal/decubitus angina | * | * | * |
| 413.1: Variant/Prinzmetal’s angina | 0.1 | 0.1 | * |
| 413.9: Other and unspecified angina pectoris (includes syncope with angina) | 0.7 | 1.3 | 1.6 |
| **Nonspecific Chest Pain and Conditions Potentially Associated With Chest Pain (CCS category may be listed as any diagnosis), % of revisits** | | | |
| 102: Nonspecific chest pain | 34.7 | 34.1 | 31.4 |
| 101: Coronary atherosclerosis and other heart disease (excluding 411.1, 413.0, 413.1, and 413.9) | 12.7 | 21.1 | 28.9 |
| 133: Other lower respiratory disease | 14.0 | 12.4 | 11.2 |
| 651: Anxiety disorders | 12.8 | 12.3 | 12.0 |
| 138: Esophageal disorders | 11.5 | 14.5 | 15.0 |
| 106: Cardiac dysrhythmias | 9.0 | 11.7 | 14.0 |
| 251: Abdominal pain | 8.7 | 7.8 | 6.2 |
| 127: Chronic obstructive pulmonary disease and bronchiectasis | 7.5 | 10.2 | 11.4 |
| 117: Other circulatory disease | 6.7 | 9.7 | 11.6 |
| 250: Nausea and vomiting | 6.6 | 6.4 | 4.8 |
| 128: Asthma | 6.6 | 6.0 | 6.0 |
| 108: Congestive heart failure; nonhypertensive | 5.3 | 8.3 | 12.5 |
| 96: Heart valve disorders | 2.1 | 2.8 | 3.7 |
| 122: Pneumonia (except that caused by tuberculosis or sexually transmitted disease) | 2.0 | 2.0 | 3.1 |
| 97: Peri-; endo-; and myocarditis; cardiomyopathy (except that caused by tuberculosis or sexually transmitted disease) | 1.8 | 2.2 | 4.3 |
| 130: Pleurisy; pneumothorax; pulmonary collapse | 2.0 | 1.5 | 1.7 |
| 140: Gastritis and duodenitis | 1.6 | 1.6 | 1.5 |
| 103: Pulmonary heart disease | 1.3 | 1.5 | 2.6 |
| 134: Other upper respiratory disease | 1.1 | 1.1 | 0.9 |
| 125: Acute bronchitis | 1.1 | 0.7 | 0.7 |
| 19: Cancer of bronchus; lung | 0.6 | 0.6 | 0.6 |
| 115: Aortic; peripheral; and visceral artery aneurysms | 0.5 | 0.6 | 1.3 |
| 107: Cardiac arrest and ventricular fibrillation | 0.2 | 0.3 | * |
| 123: Influenza | 0.2 | 0.1 | * |
| 129: Aspiration pneumonitis; food/vomitus | 0.1 | 0.2 | * |
| 100: Acute myocardial infarction (excluding 410.x0 and 410.x1 and retaining 410.x2 subsequent care) | 0.1 | 0.1 | 0.5 |
| **Unrelated Conditions (10 most common first-listed diagnosis CCS categories for each type of index visit), % of revisits** | | | |
| 205: Spondylosis; intervertebral disc disorders; other back problems | 2.6 | 2.5 | 2.3 |
| 84: Headache; including migraine | 2.5 | 2.0 | 1.6 |
| 232: Sprains and strains | 1.8 | 1.6 | 1.1 |
| 239: Superficial injury; contusion | 1.8 | 1.4 | 1.6 |
| 95: Other nervous system disorders | 1.7 | 1.7 | 1.9 |
| 211: Other connective tissue disease | 1.5 | 1.5 | 1.4 |
| 657: Mood disorders | 1.4 | 1.7 | 3.3 |
| 159: Urinary tract infections | 1.2 | 1.3 | 1.1 |
| 660: Alcohol-related disorders | 1.2 | 0.9 | 1.3 |
| 126: Other upper respiratory infections | 1.1 | 0.6 | 0.7 |
| 98: Essential hypertension | 1.0 | 2.0 | 1.4 |
| 245: Syncope | 0.9 | 1.4 | 1.4 |
| 55: Fluid and electrolyte disorders | 0.8 | 0.9 | 1.8 |
| 238: Complications of surgical procedures or medical care | 0.3 | 0.6 | 1.4 |

Abbreviations: CCS, Clinical Classifications Software (<https://hcup-us.ahrq.gov/toolssoftware/ccs/ccs.jsp>); ED, emergency department.

Note: Percentages within a category sum to greater than 100 percent because some revisits involved more than one condition.

* Suppressed because of cell size <11.

Source: Agency for Healthcare Research and Quality, Healthcare Cost and Utilization Project, State Inpatient Databases, State Emergency Department Databases, and State Ambulatory Surgery and Services Databases, 10 States (Georgia, Iowa, Maryland, Nebraska, Nevada, South Carolina, South Dakota, Tennessee, Vermont, Wisconsin), 2013 and 2014.
